# Supplementary material for: The RU486-dependent activation of the GeneSwitch system in adult muscles leads to severe adverse effects in Drosophila
Source: G3 (Bethesda). 2024 Feb 26;14(5):jkae039. doi: 10.1093/g3journal/jkae039 (PMC11075533; doi:10.1093/g3journal/jkae039)
Supplement: jkae039_Supplementary_Data [file jkae039_supplementary_data.docx]

Table S1: Sequence of primers

| **Gene** | **Forward (5'-3')** | **Reverse (5'-3')** |
| --- | --- | --- |
| RpL30 | GCAAATACTGCCTGGGCTAC | ACTTCAGTCTTGGCCAGCAT |
| RpL32 | TACAGGCCCAAGATCGTGAAG | GACGCACTCTGTTGTCGATACC |
| ND24 | AATGGTGGCGATCAACGATG | TTCAGGTCGTTCAGGATGTCC |
| ATPsynD | ACAAGAAGCTTGTGCCAGTG | TTGATCTCGGCATCCACCTG |
| Atrogin/MAFbx | TCAGCCAAGAGAACCAGAAGTG | ATGATCTCCCGCACACACTC |
| Thin/TRIM32 set2 | ATCTGTCCCATTGTGCACAC | TCACACTAATGGCGTTCGTC |
| Ref(2P)/p62 | ACACCCAGACTGCTCAAGTTG | CTCGTTGCTGAAACCCATGG |
| TER94/p97 | TGTTTGCCCAGACTTTGCAG | AAAGATCATCGTCGCCGTTG |
| Pros-alpha4 | GACGCTCGCATCATGATCAATC | GCATCGAAGCCGCCAATAAG |
| eIF4A | ATTGATGTGCAGCAGGTGTC | TTGATCGCAACACCCTTGC |
| Foxo | TCGAGTGCAATGTCGAGGAG | TGCCGGAATTGCTGCTTATG |
| Thor set2 | AAGGTTGTCATCTCGGATCCG | TGAAAGCCCGCTCGTAGATAAG |
